# Perfluorocarbon nanoparticles enhance reactive oxygen levels and tumour growth inhibition in photodynamic therapy

**DOI:** 10.1038/ncomms9785

**Published:** 2015-11-03

**Authors:** Yuhao Cheng, Hao Cheng, Chenxiao Jiang, Xuefeng Qiu, Kaikai Wang, Wei Huan, Ahu Yuan, Jinhui Wu, Yiqiao Hu

**Affiliations:** 1 State Key Laboratory of Pharmaceutical Biotechnology, Medical School, Nanjing University, Nanjing 210093, China; 2 Jiangsu Key Laboratory for Nano Technology, Nanjing University, Nanjing 210093, China

## Abstract

Photodynamic therapy (PDT) kills cancer cells by converting tumour oxygen into reactive singlet oxygen (^1^O_2_) using a photosensitizer. However, pre-existing hypoxia in tumours and oxygen consumption during PDT can result in an inadequate oxygen supply, which in turn hampers photodynamic efficacy. Here to overcome this problem, we create oxygen self-enriching photodynamic therapy (Oxy-PDT) by loading a photosensitizer into perfluorocarbon nanodroplets. Because of the higher oxygen capacity and longer ^1^O_2_ lifetime of perfluorocarbon, the photodynamic effect of the loaded photosensitizer is significantly enhanced, as demonstrated by the accelerated generation of ^1^O_2_ and elevated cytotoxicity. Following direct injection into tumours, *in vivo* studies reveal tumour growth inhibition in the Oxy-PDT-treated mice. In addition, a single-dose intravenous injection of Oxy-PDT into tumour-bearing mice significantly inhibits tumour growth, whereas traditional PDT has no effect. Oxy-PDT may enable the enhancement of existing clinical PDT and future PDT design.

## Abstract (web summary)

Photodynamic therapy is used in cancer treatment and generates reactive oxygen species to kill tumour cells but is limited by the availability of oxygen. Here, the authors modify a photodynamic sensitiser so that it produces excess oxygen species and show enhanced tumour cell killing *in vitro* and *in vivo*.

## Graphical abstract

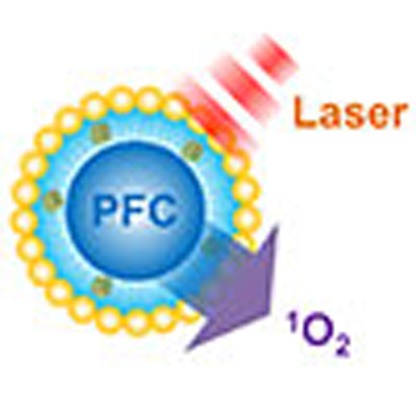

Photodynamic therapy (PDT) depends on the ability of photosensitizers (PS) to transfer energy from lasers to tumour-dissolved oxygen (O_2_) to generate cytotoxic singlet oxygen (^1^O_2_) for cancer treatment^1^,^2^. However, the effectiveness of PDT is impaired by an inadequate oxygen supply in tumours^3^,^4^. In most solid tumours, hypoxia is common because the oxygen supply is reduced by disturbed microcirculation and deteriorated diffusion^5^. Moreover, PDT worsens hypoxia through oxygen consumption and vascular shutdown effects^6^. Low oxygen content can reduce the photodynamic efficacy of PS, preventing PDT from achieving its full therapeutic potential^7^.

Traditional methods have attempted to optimize tumour oxygenation to ensure PDT efficacy. For example, dividing irradiation into light–dark circles^8^ and extending irradiation with a low fluence rate^9^ have both been investigated as techniques for better tumour reoxygenation by the blood. However, these approaches only affect PDT-induced oxygen depletion, whereas the pre-existing hypoxia cannot be reversed; moreover, vascular shutdown due to PDT would also result in severe hypoxia^10^. Hyperbaric oxygen inhalation has also been used to actively increase the level of tumour oxygen^11^,^12^,^13^,^14^. However, vascular damage during PDT still prevents further oxygenation from hyperbaric blood^15^; moreover, the potential toxic effects of excessive oxygen are an impediment to its clinical use^16^,^17^. To our knowledge, no existing techniques can effectively reverse the tumour oxygen content during PDT. Therefore, optimizing the efficacy with limited oxygen is of great importance for photodynamic therapy.

To address this challenge, herein we load photosensitizer into perfluorocarbon nanodroplets to develop a novel oxygen self-enriched photodynamic therapy (Oxy-PDT). Because of its high oxygen capacity^18^, perfluorocarbon can maintain a higher oxygen content than the tumour matrix at a given oxygen partial pressure (Supplementary Fig. 1). Thus, although the tumour oxygen content remains limited during PDT, sufficient O_2_ can always be enriched in the PFC droplet for photodynamic consumption by the loaded PS, thus obtaining improved efficacy (Fig. 1a). This type of enhancement is possible regardless of pre-existing hypoxia, photodynamic consumption, or vascular damage; moreover, it has been reported that the ^1^O_2_ lifetime in perfluorocarbon is much longer than in the cellular environment or in water^19^, which results in long-lasting photodynamic effects. Therefore, Oxy-PDT might help PS to achieve its full therapeutic potential. In this study, we assessed the therapeutic efficacy of Oxy-PDT as a novel form of PDT in cancer models. First, we studied the photodynamic effect of Oxy-PDT on the generation of ^1^O_2_ and its cytotoxicity by incubating tumour cells with Oxy-PDT, which were then irradiated with laser beams. Next, we assessed the effect of Oxy-PDT on tumour growth by intratumoural injections *in vivo*. Last, we examined the passive targeting of Oxy-PDT by intravenous injections *in vivo*. We envision that this new approach may guide improvements in the clinical use of PDT.

## Results

### Synthesis of the Oxy-PDT agent

**Figure 1:**
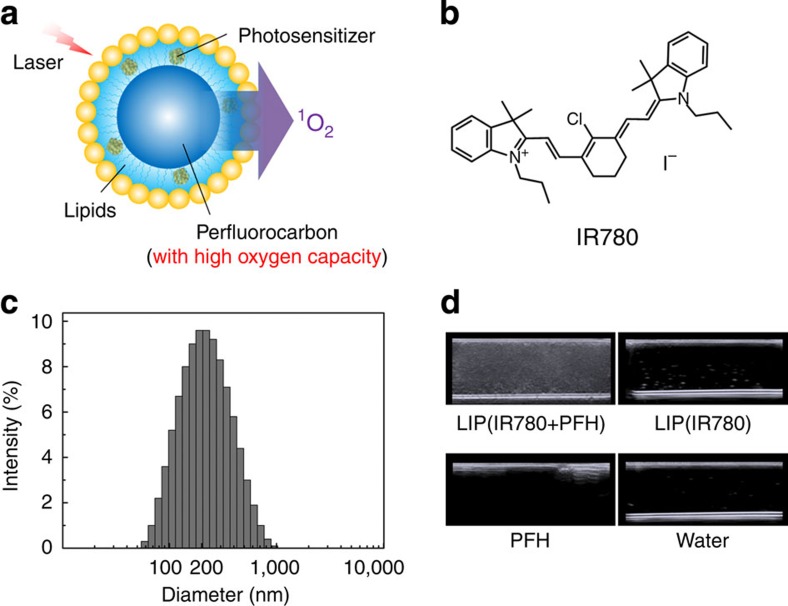
Characterization of the Oxy-PDT agent. (**a**) Structure and design of the Oxy-PDT agent. Photosensitizer and perfluorocarbon are coencapsulated by lipids. Photosensitizer is uniformly dispersed inside the lipid monolayer and PFC in the core of the nanoparticle. When irradiated by laser, PS transfers energy to the oxygen enriched in PFH, producing ^1^O_2_, resulting in enhanced tumour inhibition. (**b**) Structure of IR780. (**c**) Dynamic light scattering of the Oxy-PDT agent. (**d**) Ultrasound images of the Oxy-PDT agent and other groups tested in 5-ml plastic test tubes.

The near-infrared photosensitizer IR780 (Fig. 1b) and perfluorohexane (PFH) were used in this manuscript to prepare the Oxy-PDT agent LIP(IR780+PFH). IR780 is uniformly dispersed in a lipid monolayer (composed of lecithin, cholesterol, and DSPE-PEG2000), and the average diameter of the resulting nanodroplets is 200 nm (Fig. 1c). As the Oxy-PDT agent is irradiated by a near-infrared (NIR) 808-nm laser, IR780 transfers energy to the oxygen enriched in the PFH, producing cytotoxic singlet oxygen (Fig. 1a). The existence of IR780 in the Oxy-PDT agent was confirmed by the absorption peak in the NIR region (Supplementary Fig. 2). The existence of PFH in the Oxy-PDT agent was confirmed by the contrast enhancement in the ultrasound image, whereas LIP(IR780), pure PFH, and water produced blank echo signals (Fig. 1d). PFH in Oxy-PDT agent was also confirmed by GC-Mass (Supplementary Fig. 2b,c). The stability and self-quenching capability of the Oxy-PDT agent were then evaluated (Supplementary Fig. 3). The results indicate that Oxy-PDT can increase the dark stability of IR780 and reduce its self-quenching.

### Characterization of photodynamic efficacy

**Figure 2:**
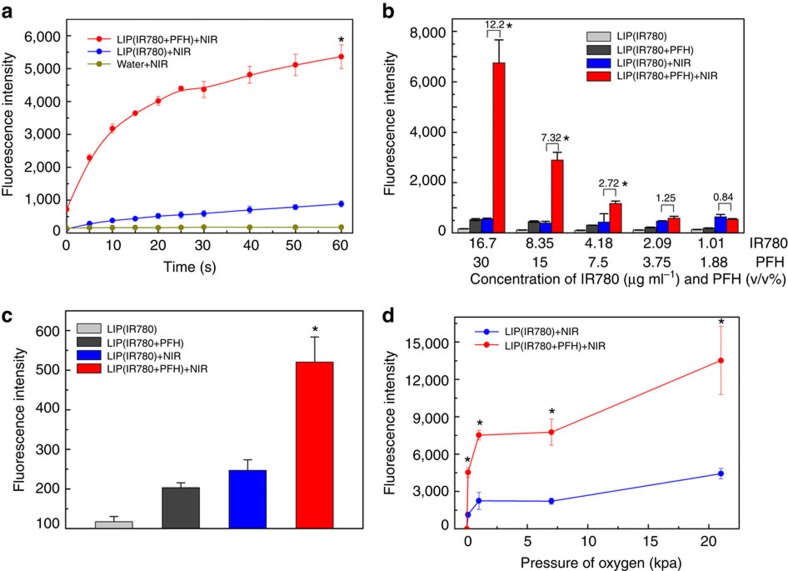
^1^O_2_ production of different samples under NIR laser irradiation as determined by the accumulated fluorescence intensity of oxidized SOSG. (**a**) ^1^O_2_ production of LIP(IR780+PFH) (16.7 μg ml^−1^ IR780, 30 v/v% PFH), LIP(IR780) (16.7 μg ml^−1^ IR780), and water under different laser irradiation exposures. Values are the means±s.d. (*n*=3, *P<0.05, versus LIP(IR780)+NIR, two-sided Student's *t*-test); (**b**) ^1^O_2_ production in different diluted samples. The concentrations of IR780 and PFH varied from 16.7 to 1.01 μg ml^−1^ and 30% to 1.88% (v/v%), respectively. Values are the means±s.d. (*n*=3, *P<0.05, versus LIP(IR780)+NIR, two-sided Student's t-test). Irradiation was performed by an 808 nm laser (2 W cm^−2^) for 20 s. (**c**) ^1^O_2_ production of LIP(IR780+PFH) with a low concentration of IR780 and a high concentration of PFH (1.01 μg ml^−1^ IR780, 30 v/v% PFH). Values are the means±s.d. (*n*=3). Irradiation was performed by an 808-nm laser (2 W cm^−2^) for 20 s, (*P<0.05, versus LIP(IR780)+NIR, two-sided Student's *t*-test); (**d**) ^1^O_2_ production after 20-s irradiation in different hypoxic conditions (0.1, 1, 7, or 21 kPa O_2_), for LIP(IR780+PFH) (16.7 μg ml^−1^ IR780, 15 v/v% PFH) and LIP(IR780) (16.7 μg ml^−1^ IR780). Values are the means±s.d. (*n*=3, *P<0.05, versus LIP(IR780)+NIR, two-sided Student's *t*-test).

We compared the ^1^O_2_ generation by Oxy-PDT with traditional PDT, as determined by the fluorescence intensity of oxidized SOSG (an indicator of ^1^O_2_). In the presence of ^1^O_2_, the oxidation of SOSG results in increased fluorescence and therefore provides a means of monitoring ^1^O_2_ production. We measured the varying extent of fluorescence gain in water in the presence of LIP(IR780+PFH) or LIP(IR780) irradiated by an 808-nm laser for 5-s intervals (Fig. 2a). The presence of LIP(IR780+PFH) resulted in the highest rate of fluorescence gain, and the total accumulation was also significantly higher than in the presence of LIP(IR780), which resulted in only a marginal fluorescence gain (*P*<0.05, two-sided Student's *t*-test). The singlet oxygen quantum yields (Ф_so_) of both LIP(IR780) and LIP(IR780+PFH) were also calculated (Supplementary Table 1). The Ф_so_ value of LIP(IR780+PFH) is higher than for LIP(IR780) or IR780, indicating that the Oxy-PDT platform can increase the quantum yield of photosensitizer. Moreover, this phenomenon can also be achieved using other photosensitizers, suggesting that the enhancement is not specific to IR780 (Supplementary Fig. 4).

To determine whether such fluorescence gain is a result of the presence of PFH, we diluted LIP(IR780+PFH) and LIP(IR780) to a series of solutions with concentrations of PFH ranging from 30 v/v% to 1.88 v/v% (Fig. 2b). The dilution caused a marked relative reduction in the fluorescence between 20 s continually irradiated LIP(IR780+PFH) and LIP(IR780), from 12.2- to 0.84-fold. Moreover, the enhancement was proven to be independent of IR780 concentration (Supplementary Fig. 5), indicating that the presence of PFH is a key factor in the enhanced PDT effect. Then, when we added enough PFH (resulting in 30 v/v% PFH) to the diluted LIP(IR780+PFH), the relatively reduced fluorescence recovered (Fig. 2c), confirming the participation of PFH in the generation of ^1^O_2_.

We then tested the photodynamic efficacy of both LIP(IR780) and LIP(IR780+PFH) in different hypoxic conditions (Fig. 2c). The samples were exposed to different atmospheres (containing 0.1, 1, 7 and 21 kPa O_2_) and then received 20 s irradiation. The results suggested that LIP(IR780+PFH) significantly enhanced ^1^O_2_ production under different oxygen pressures. Meanwhile, LIP(IR780+PFH) in extremely hypoxic conditions (0.1 kPa O_2_) showed higher ^1^O_2_ generation than LIP(IR780) in non-hypoxic conditions (21 kPa O_2_), indicating that during Oxy-PDT treatment, the PFC can successfully enrich oxygen to accelerate ^1^O_2_ generation.

### Cell assays

**Figure 3:**
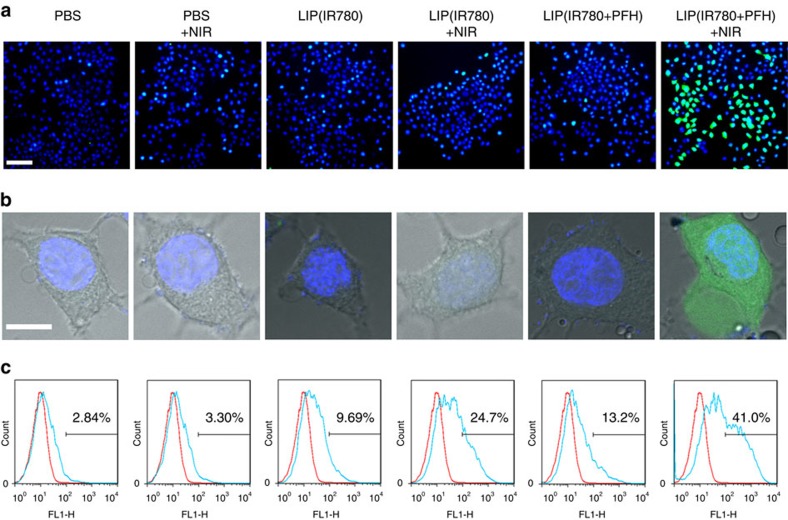
Enhanced ^1^O_2_ production by Oxy-PDT in cells. (**a**) Cells were treated with different agents and then exposed to an 808-nm laser (2 W cm^−2^ for 20 s). ROS generation was detected using carboxy-H_2_DCFDA. Confocal images showing green fluorescence indicate positive staining for ROS; the positions of the cells are shown by blue fluorescence indicative of nuclear counterstaining with Hoechst 33342 (scale bar, 50 μm). (**b**) Magnified images (scale bar, 10 μm). (**c**) Flow-cytometry analysis of ROS generation in cells treated with different agents and then exposed to an 808-nm laser (2 W cm^−2^ for 20 s) detected using carboxy-H_2_DCFDA.

Assays were performed on both MCF-7 human breast cancer cells and CT26 murine colon adenocarcinoma cells. First, we examined the production of reactive oxygen species (ROS) such as ^1^O_2_ from Oxy-PDT and traditional PDT in live cells using carboxy-H_2_DCFDA as a fluorogenic marker for ROS. Carboxy-H_2_DCFDA permeates cell membranes to be oxidized, emitting bright green fluorescence in the presence of ^1^O_2_. The resulting oxidatively stressed cells showed green fluorescence, indicating increased ^1^O_2_ gain. We treated cells with PBS, LIP(IR780) or LIP(IR780+PFH), with and without 20 s continual 808-nm laser irradiation. Without laser irradiation, MCF-7 cells treated with PBS, LIP(IR780) or LIP(IR780+PFH) showed negligible fluorescence (Fig. 3a,b). After irradiation, cells treated with LIP(IR780+PFH) showed significant green fluorescence, whereas cells treated with PBS and LIP(IR780) still showed negligible fluorescence. Similar results can also be observed in treated CT26 cells (Supplementary Fig. 6). To verify the results, flow cytometry was used to analyse the percentage of cells stained with green fluorescence (Fig. 3c). Cells treated with PBS, LIP(IR780) and LIP(IR780+PFH) showed a relatively low percentage (2.84%, 9.69% and 13.2%, respectively) of green fluorescent cells, whereas cells treated with LIP(IR780) showed an increased percentage (24.7%) after irradiation. More notably, the percentage (41.0%) in cells treated with LIP(IR780+PFH) and an 808-nm laser was much higher than in cells treated with LIP(IR780), indicating the better performance of LIP(IR780+PFH) as the ^1^O_2_ producer.

**Figure 4:**
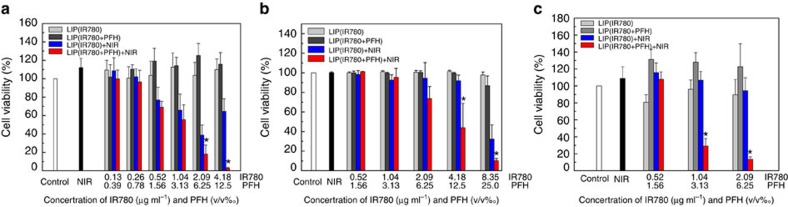
Enhanced cytotoxicity by Oxy-PDT agents in normal and hypoxic conditions. Cells were exposed to 2 W cm^−2^ of an 808-nm NIR laser for 20 s, and viability was measured by the CCK-8 assay. (**a**) MCF-7 cells; (**b**) CT-26 cells; (**c**) CT-26 cells in hypoxic conditions. Values are the means±s.d. (*n*=3, *P<0.05 versus LIP(IR780)+NIR, two-sided Student's *t*-test).

After comparing their performances as ^1^O_2_ producers, we tested Oxy-PDT and traditional PDT for their photodynamic effects on inducing cell death. Without laser irradiation, we found no significant cytotoxicity in MCF-7 cells treated with LIP(IR780) or LIP(IR780+PFH) (Fig. 4a). After 20 s continual irradiation by an 808-nm laser, we detected a reduction in the viability of cells treated with both LIP(IR780) and LIP(IR780+PFH). It is notable that the reductions in viability were higher in cells treated with LIP(IR780+PFH) than in cells treated with LIP(IR780) when the concentrations of IR780 were higher than 2.09 μg ml^−1^ (*P*<0.05, two-sided Student's *t*-test). Similar results were also observed in CT26 murine colon adenocarcinoma cells (Fig. 4b). In addition, cell death induced by photothermal effects was not evident in the minimal IR780 and irradiation dosages (Supplementary Fig. 7a).

The effects of Oxy-PDT in hypoxia were also studied (Fig. 4c). CT26 cells were placed in a hypoxic transparent box for 4 h before irradiation. Then, laser irradiation and a further 1-h incubation were performed. Interestingly, in hypoxic conditions, Oxy-PDT still maintained superior cytotoxicity to traditional PDT (*P*<0.05, two-sided Student's *t*-test), indicating that Oxy-PDT can improve the efficacy of PDT in both normal and hypoxic conditions.

### *In vivo* PDT using intratumoural injection

**Figure 5:**
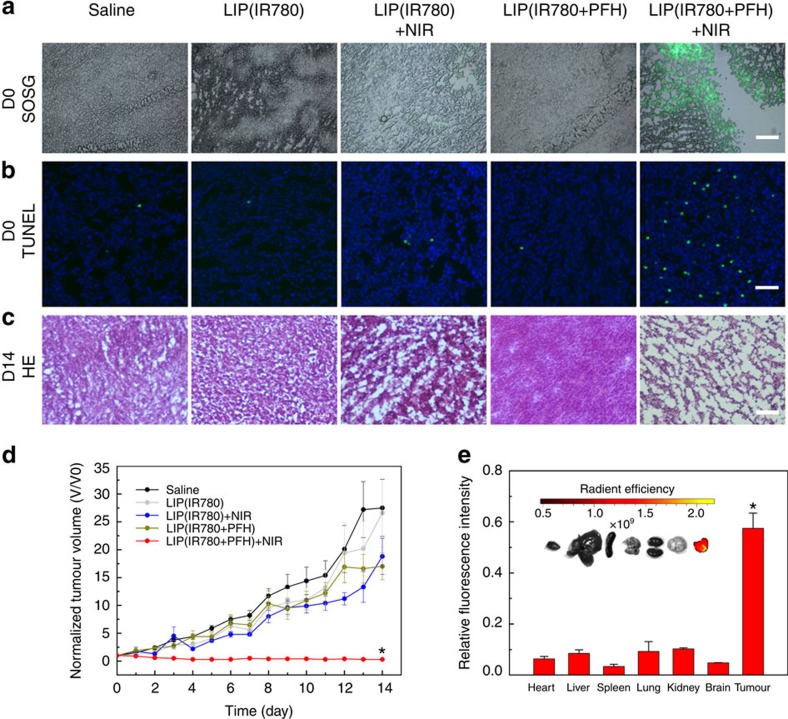
*In vivo* photodynamic therapy of Oxy**-PDT by intratumoural injection in a subcutaneous tumour model. (**a**) SOSG staining in tumour sections for ^1^O_2_ determination (scale bar, 100 μm). Before sectioning, a mixture of SOSG with saline, LIP(IR780), or LIP(IR780+PFH) was injected into the tumours, followed by laser irradiation. (**b**) TUNEL staining for apoptosis in tumour sections from each group to determine the effectiveness of Oxy-PDT. DAPI counterstaining indicates the tumour nuclear region (scale bar, 50 μm). (**c**) H&E staining for pathological changes in tumour sections from each group to determine the effectiveness of Oxy-PDT (scale bar, 50 μm). (**d**) Changes in tumour volumes used to assess the effectiveness of Oxy-PDT in tumour-bearing mice by intratumoural injection. Values are the means±s.e.m. (*n*=6, *P<0.05 versus LIP(IR780)+NIR, two-sided Student's *t*-test). (**e**) Biodistribution of intratumourally injected LIP(IR780+PFH) in organs of mice at 14 days after initial treatment. Organs were isolated and quantified by near-infrared imaging (based on IR780 content). Values are the means±s.e.m. (*n*=6).

The *in vivo*^1^O_2_ generation of both LIP(IR780) and LIP(IR780+PFH) was measured. SOSG was also used as an indicator because of its green fluorescence after oxidization by the generated ^1^O_2_. Without laser irradiation, tumours treated with LIP(IR780) or LIP(IR780+PFH) showed negligible fluorescence. After laser irradiation, tumours treated with LIP(IR780+PFH) showed significant green fluorescence, whereas tumours treated with LIP(IR780) showed weak fluorescence (Fig. 5a). This result indicated that higher ^1^O_2_ generation by Oxy-PDT can also be achieved *in vivo*.

We further compared the efficacy of Oxy-PDT and traditional PDT *in vivo* on CT26 tumour-bearing mice via intratumoural injections. The mice were divided into five groups, including a saline control group and LIP(IR780) and LIP(IR780+PFH) groups with and without 808-nm laser irradiation. To avoid the additional efficacy induced by photothermal effects, the temperature of the tumour during irradiation was controlled by dividing the irradiation time into two consecutive 10 s applications with a 1-min interval in between (Supplementary Fig. 7b). We first evaluated the efficacy of the treatment in terms of tumour cell apoptosis by performing TUNEL staining on histological sections from the different treatment groups immediately after drug administration. We found a greater number of apoptotic cells in histological sections from the group receiving LIP(IR780+PFH) and irradiation (Fig. 5b), which suggested the successful destruction of tumour cells by Oxy-PDT.

We then assessed the photodynamic effects of each group by monitoring the tumour volumes over a period of 2 weeks (Fig. 5d, Supplementary Data 1). The tumour volumes in mice receiving LIP(IR780+PFH) with 808-nm laser irradiation were significantly inhibited, whereas no significant differences in tumour volumes were found between the saline control group and other treatment groups, including LIP(IR780+PFH) and LIP(IR780) with and without laser irradiation. We then performed H&E staining on tissue sections from each group at 14 days after treatment. We found that most tumour cells were severely damaged or destroyed in mice treated with LIP(IR780+PFH) and irradiation by an 808-nm laser (Fig. 5c). In the other groups, there was incomplete tumour cell death, suggesting the successful destruction of tumour cells by Oxy-PDT. At 14 days after treatment, a biodistribution study of the mice receiving only the intratumoural injection of LIP(IR780+PFH) and irradiation showed the presence of IR780 only in the tumour tissues (Fig. 5e), suggesting that IR780 remained primarily in the tumour mass after administration and that the diffusion from tumour tissue to other tissues was negligible.

### *In vivo* PDT using intravenous injection

**Figure 6:**
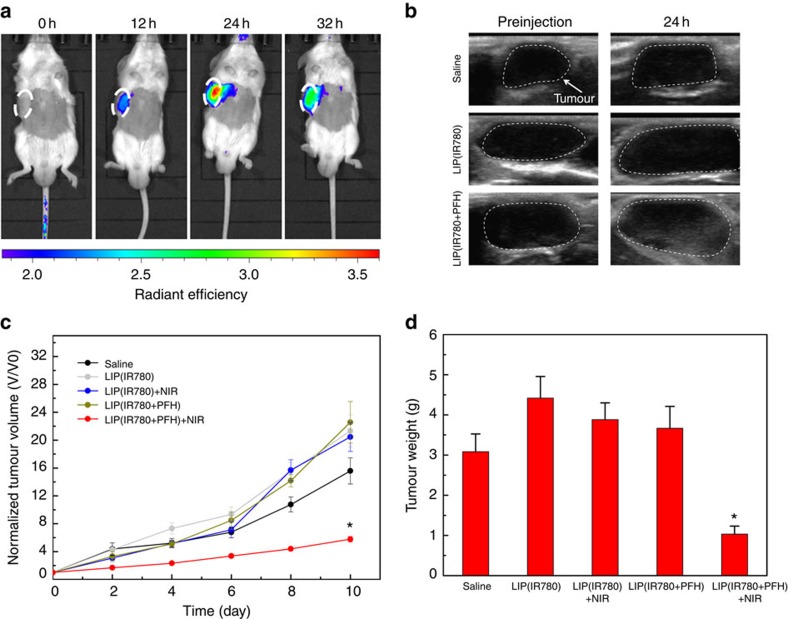
*In vivo* photodynamic therapy of Oxy**-PDT by intravenous injection in a subcutaneous tumour model. (**a**) Near-infrared imaging of tumour accumulation of IR780 in tumour-bearing mice after intravenous injection of LIP(IR780+PFH). Images were taken at 0, 12, 24 and 32 h postinjection. Tumours are circled with white dashed lines. (**b**) Ultrasound imaging of tumours on mice before (0 h) and after intravenous injection (24 h) of LIP(IR780+PFH) (0.2 ml, 60 μg ml^−1^ IR780, 10 v/v% PFH), LIP(IR780) (0.2 ml, 60 μg ml^−1^ IR780), and saline. Tumours are circled with white dashed lines. (**c**) Changes in tumour volumes used to assess the effectiveness of Oxy-PDT in tumour-bearing mice by intravenous injection. Treatments were performed only once. Values are the means±s.e.m. (*n*=6, *P<0.05 versus LIP(IR780)+NIR, two-sided Student's *t*-test). (**d**) Average weights of tumours at day 10. Mice were killed, and tumours were isolated for weighing. Values are the means±s.e.m. (*n*=6, *P<0.05 versus LIP(IR780)+NIR, two-sided Student's *t*-test).

After the study of intratumoural injection, we examined the efficacy of Oxy-PDT by intravenous injection. Because of the enhanced permeability and retention effect, nanoparticles in the range of 10–400 nm can accumulate in tumour tissue at levels 70 times higher than in normal tissue^20^,^21^. Accordingly, LIP(IR780+PFH) with a mean particle size of 200 nm showed passive tumour targeting, as demonstrated by the NIR (Supplementary Fig. 8) and the ultrasound imaging of tumour accumulation after intravenous injection. The tumour accumulation of IR780 reached its maximum at 24 h after intravenous injection (Fig. 6a); meanwhile, ultrasound imaging confirmed the existence of PFH in the tumours at 24 h (Fig. 6b). Therefore, 24 h after intravenous injection was chosen for 808-nm laser irradiation.

Mice were divided into five groups, including the saline control group and the LIP (IR780) and LIP (IR780+PFH) groups with and without laser irradiation. To avoid the confounding effects induced by photothermal radiation, the temperature of the tumour during irradiation was controlled within a safe range by dividing the irradiation time into two consecutive 10-s applications with a 1-min interval (Supplementary Fig. 7b). Monitoring the change in tumour volume as a function of time after treatment revealed a significant reduction in the tumour growth rate of mice receiving LIP(IR780+PFH) and laser irradiation (*P*<0.05, versus LIP(IR780)+NIR, two-sided Student's *t*-test, Fig. 6c, Supplementary Data 2). No significant reduction was found in the tumour growth rate of mice receiving LIP(IR780) and laser irradiation. At the end point of day 10, the tumours from each mouse were excised and weighed (Fig. 6d). The mean tumour weight in the mice receiving LIP(IR780+PFH) and laser irradiation was approximately fourfold lower than in mice receiving LIP(IR780) and laser irradiation (*P*<0.05, two-sided Student's *t*-test), suggesting the improved efficacy of Oxy-PDT.

Although the tumours were significantly inhibited, they were not completely regressed as a result of intravenous injection plus laser irradiation. Indeed, significant tumour inhibition was obtained by a mere single-dose injection and 20-s irradiation, compared with traditional PDT, in which the dosage was too low to show any therapeutic effect. In light of the current results, further optimization of administration frequency is warranted to exploit the full potential of Oxy-PDT-enhanced photosensitizer as a new PDT clinical approach

## Discussion

In this study, we demonstrate a novel Oxy-PDT platform for achieving enhanced efficacy. PDT depends strongly on ^1^O_2_ to kill cancer cells, and thus the ^1^O_2_ generation rate determines PDT efficacy. In the Oxy-PDT agent, both PS and oxygen are enriched in the nanodroplet, and thus the oxygen available for the photodynamic reaction are increased compared with traditional PDT in the tumour matrix. Moreover, the lifetime of ^1^O_2_ varies with the polarity of the solvent: the half-life of ^1^O_2_ is ∼6 × 10^−7^ s in the cellular environment, 5 × 10^−6^ s in water, and 5 × 10^−2^ s in PFH^19^. Therefore, by using a PFC nanodroplet as the PS carrier, the lifetime of ^1^O_2_ is extended. These phenomena may be two major reasons that Oxy-PDT can enhance the reactive oxygen level.

NIR dyes (such as IR780) typically have a lower ^1^O_2_ quantum yield (Ф_so_) than visible dyes because the wavelength of NIR light (>800 nm) is longer than the wavelength of visible light and may have insufficient energy to excite oxygen to its singlet state^1^,^2^,^22^. However, in this study, Ф_so_LIP(IR780+PFH) was found to be higher than Ф_so_LIP(IR780), demonstrating that the Oxy-PDT platform can increase the ^1^O_2_ quantum yield of NIR dyes. Furthermore, our results showed that Oxy-PDT could increase the dark stability of IR780 and reduce its self-quenching.

The results also showed a higher therapeutic efficacy of Oxy-PDT than traditional PDT both *in vitro* and *in vivo*. With direct injection into tumours, the *in vivo* studies showed complete tumour growth inhibition in Oxy-PDT mice treated with a low photosensitizer dosage and 20-s laser irradiation, whereas traditional PDT showed negligible tumour inhibition. These results suggested that in Oxy-PDT, PFH can help the photosensitizer achieve improved effects. The ultraviolet–visible absorption of the NIR dye is reduced after laser irradiation, possibly due to the degradation of the NIR dye by exposure to ^1^O_2_. Although the degradation products might also be toxic to cells, the enhanced tumour growth effect of LIP(IR780+PFH) is still attributed to PFH because the possible degradation product is also present in LIP(IR780). Interestingly, with only a single-dose intravenous injection into tumour-bearing mice, Oxy-PDT still exhibited significant tumour inhibition, indicating that passive targeting plays an important role in the therapeutic efficacy. It should be noted that in all our experiments, the influence of photothermal therapy has been excluded to examine only photodynamic effects. Moreover, previous studies have already confirmed the high oxygen content in perfluorocarbons, which are used as artificial blood in clinical applications and can be used as ultrasonography^23^ and MRI^24^,^25^ contrast agents to monitor the behaviour of Oxy-PDT agents *in vivo.*

As previously discussed, Oxy-PDT is considered to be the first PDT design that can realize high efficacy in hypoxic conditions. This Oxy-PDT approach with self-enriching oxygen offers a simple yet effective treatment option for cancer patients. We have provided a proof of concept for the PFC/photosensitizer-loaded emulsion as an agent of improved PDT *in vivo* and anticipate its wide clinical application after dose optimization.

## Methods

### Chemicals and reagents

Lecithin and cholesterol were purchased from Aladdin Industrial Corporation, and DSPE-PEG2000 was obtained from A.V.T. Pharm. Ltd. (Shanghai, China). The 99% perfluorohexane was purchased from Bailingwei Tech Co., Ltd. (Beijing, China). IR780 was purchased from Sigma-Aldrich Chemical Corporation (St Louis, MO, USA). Singlet Oxygen Sensor Green was obtained from Molecular Probes, Inc. Carboxy-H2DCFDA was purchased from Invitrogen (USA). Cell counting kit-8 (CCK-8) was supplied by Dojindo Laboratories (Japan). MCF-7 and CT-26 cells were purchased from the Cell Bank of Shanghai Institutes for Biological Sciences, Chinese Academy of Sciences (Shanghai, China). All of the BALB/c mice were purchased from Yangzhou University Medical Centre (Yangzhou, China). All of the chemicals were used as supplied without further purification.

### Synthesis of the Oxy-PDT agent

First, 24.65 mg lecithin, 4.28 mg cholesterol, 3.79 mg DSPE-PEG2000, and the specified IR780 were dissolved in 5 ml dichloromethane. The dichloromethane was removed from a 25-ml round flask by rotary evaporation to form lipid films. Using 10-min sonication, 1.4 ml pure water was added, and the film was peeled off. Next, 0.6 ml PFH was added gradually under high-speed dispersion (IKA, T25, German) at 24,000 r.p.m. in an ice bath for 10 min to form 2 ml LIP(IR780+PFH) (30 v/v% PFH). LIP(IR780) can be formed by the addition of pure water instead of PFH.

### Detection of singlet oxygen *in vitro*

First, 0.1 ml samples and 0.02 ml of 50 μM SOSG were mixed in black 96-well plates (Costar). After irradiation (808-nm, 2 W cm^−2^), the oxidized SOSG was quantified by measuring the fluorescence intensity (excited at 504 nm and measured at 525 nm) using a multifunctional microplate reader (Safire, TECAN). As for the production of singlet oxygen in hypoxia, samples were kept in transparent box with different hypoxic air (0.1, 1, 7 or 21 kpa O_2_). After laser irradiation (808-nm, 2 W cm^−2^), the oxidized SOSG was quantified by measuring the fluorescence intensity. All operations were performed without light exposure. The experiments for each group were run in triplicate.

The MCF-7 cells were seeded with a density of 2 × 10^5^ per well in 12-well plates. After the cells were incubated for 24 h, the medium was replaced with 700 μl fresh culture medium. Then, 350 μl PBS, LIP(IR780) or LIP(IR780+PFH) was added to the well. The cells were further incubated for 30 min at 37 °C and 5% CO_2_. The final concentration of IR780 was 4 μg ml^−1^. After washing once with PBS, the cells were incubated with 600 μl carboxy-H_2_DCFDA (25 μM) for 10 min. Subsequently, the cells were washed once with PBS and irradiated by an 808-nm laser (2 W cm^−2^) for 20 s per well. Then, the cells were fixed by 4% formaldehyde polymer for 10 min and labelled with 600 μl Hoechst 33342 (1 μM) for 5 min. Finally, the cells were replaced with 1 ml PBS. The fluorescence emission spectrum of carboxy-DCF (Ex/Em=495/529 nm) and Hoechst 33342 (Ex/Em=350/461 nm) were immediately captured on a confocal fluorescence microscope (OLYMPUS FV1000).

### Flow cytometry

The MCF-7 cells were seeded with a density of 5 × 10^5^ per well in 12-well plates. After incubation for 24 h, the medium was replaced with 1.4 ml fresh culture medium. Then, 700 μl PBS, LIP(IR780), or LIP(IR780+PFH) was added to each well, as appropriate. The cells were further incubated for 50 min at 37 °C in an atmosphere containing 5% CO_2_. The final concentration of IR780 was 4 μg ml^−1^. After washing once with PBS, the cells were incubated with 550 μl carboxy-H_2_DCFDA (25 μM) for 10 min. Subsequently, the cells were washed once with PBS and irradiated by an 808-nm laser (2 W cm^−2^) for 20 s per well. The cells were then centrifuged, resuspended in 500 μl PBS, and analysed by flow cytometry (FACS-Calibur, BD Corp.). Green fluorescence was collected on the FL1 channel due to intracellular carboxy-DCF. Data were obtained and analysed using the CELL QUEST and FLOWJO programs.

### Cytotoxicity

The MCF-7 or CT26 cells were seeded into 96-well plates at a density of 4 × 10^4^ cells per well. After incubation for 24 h, the cells were treated with LIP(IR780) or LIP(IR780+PFH) at different concentrations (final concentrations of 20 μl samples mixed with 100 μl culture medium). For cytotoxicity in hypoxia, the cells were put into a GENbox Jar, where GENbox anaer and anaerobic atmosphere indicator were included. As the oxygen was consumed by GENbox anaer, the indicator changed its colour from blue to colourless. The Jar was approximately oxygen-free 4 h later. Then, the cells were immediately irradiated by an 808-nm laser (2 W cm^−2^) for 20 s per well. After co-incubation for 2 h, the drugs were removed, and fresh culture medium was added. After further incubation for 24 h, a mixed solution consisting of CCK-8 (10 μl) and fresh culture medium (100 μl) was added to each well and incubated for an additional 2 h at 37 °C and 5% CO_2_. Finally, the absorbance was measured at 450 nm using the microplate reader. Cells without any drugs or NIR irradiation were used as a negative control.

### Animals

BALB/C male mice aged 4–6 weeks were purchased from Yangzhou University Medical Centre (Yangzhou, China) and were used in accordance with the regulations of the Institutional Animal Care and Use Committee (IACUC) of Nanjing University. During the study, animals were observed for any clinically relevant abnormalities daily or once every other day. If any animal was moribund due to treatment-associated toxicity, tumour over-growth (⩾3,000 mm^3^), then the loss of 20% of body weight relative to the start of the study, or the appearance of large or open ulceration in the xenograft before scheduled killing, it was killed by CO_2_ inhalation. In several instances, however, tumour volumes were allowed to exceed the stated limit of 3,000 mm^3^ because the affected mice appeared otherwise healthy and had lost <5% of their original body weight. This implementation of the protocol has been confirmed by NJU-IACUC. Hairs on the flanks of the mice were removed before further treatments. Tumours were first developed in BALB/C mice by subcutaneously implanting 1 × 10^7^ CT26 cells suspended in 100 μl of serum-free DMEM in the lower flanks of the mice. When the tumour volume reached ∼200 mm^3^, the tumour mass was removed and cut into small pieces of approximately 2–6 mm^3^, which were subcutaneously implanted into other mice.

### Detection of singlet oxygen *in vivo*

Tumours were developed in 4- to 6-week-old BALB/C male mice. When the tumour volume reached ∼1,000 mm^3^, the following study was performed. A mixture of 25 μl SOSG (50 μM), 25 μl saline, and LIP(IR780) (156 μg ml^−1^ IR780) or LIP(IR780+PFH) (156 μg ml^−1^ IR780, 30 v/v% PFH) was directly injected into tumours. Subsequently, laser treatment (with an 808-nm laser (2 W cm^−2^) for two consecutive exposures of 10 s each, with a 1-min interval between the two irradiations) was performed on the LIP(IR780)+NIR and LIP(IR780+PFH)+NIR groups to generate ^1^O_2_. Then, tumours from the five groups were collected and cryosectioned onto slides at a 7-μm thickness. The fluorescence emission of the oxidized SOSG (Ex/Em=504/525 nm) was captured by fluorescence microscope.

### *PDT* in tumour-bearing mice by intratumoural injection

PDT treatments were then performed on 4- to 6-week-old BALB/C male mice 7 days after inoculation of the 2–6 mm^3^ tumour pieces. The groups were as follows: group 1: saline; group 2: LIP(IR780); group 3: LIP(IR780)+NIR; group 4: LIP(IR780+PFH); group 5: LIP(IR780+PFH)+NIR. First, 50 μl of LIP(IR780) (156 μg ml^−1^ IR780), LIP(IR780+PFH) (156 μg ml^−1^ IR780, 30 v/v% PFH) or saline alone was directly injected into the tumour mass. Then, laser treatment was immediately performed on groups 3 and 5 by irradiating the tumour regions with an 808-nm laser (2 W cm^−2^) for two consecutive exposures of 10 s each, with a 1-min interval between exposures for tumour cooling. At day 0, tumours from the five groups were cryosectioned at 5-μm thickness onto slides and stained with TUNEL (Roche, Basel, Switzerland) according to the manufacturer's instructions. Tumour size was measured daily using a vernier calliper for 14 days after the first PDT treatment. The maximum width (*X*) and length (*Y*) of the tumours were measured, and the tumour volumes (*V*) were calculated using the formula *V*=(*X*^2^*Y*)/2. Changes in tumour volume as a function of time were determined for each mouse by normalizing the tumour volume at day *X* to their respective tumour volume at day 0 after treatment. Mice were randomly selected in each group, and the tumours were photographed at days 7 and 14. At 14 days after treatment, the tumour, heart, liver, spleen, lung, kidney and brain tissues of mice from group 5 were collected and detected under *in vivo* small animal imaging for the biodistribution study. Tumours from the five groups were cryosectioned at 7-μm thickness onto slides and stained with H&E according to the manufacturer's instructions.

### *PDT* in tumour-bearing mice by intravenous injection

The CT26 subcutaneous tumours were first developed in the mice as described above. PDT treatment was then performed at 8 days after inoculation of the tumour pieces. The average initial tumour volume was 144.4 mm^3^. The testing groups were as follows: group 1: saline; group 2: LIP(IR780); group 3: LIP(IR780)+NIR; group 4: LIP(IR780+PFH); group 5: LIP(IR780+PFH)+NIR. First, 200 μl of LIP(IR780) (60 μg ml^−1^ IR780), LIP(PFH+IR780) (60 μg ml^−1^ IR780, 10 v/v% PFH) or saline alone was intravenously injected. NIR images of IR780 accumulation in the tumours of the mice in the LIP(IR780+PFH) group were taken at 0, 12, 24 and 32 h, and ultrasound images of the tumour accumulation of PFH were taken pre- and 24-h postinjection. Twenty-four hours later, laser treatment was performed on groups 3 and 5 by irradiating the tumour region with an 808-nm laser (2 W cm^−2^) for two consecutive exposures of 10 s each, with a 1-min interval between the two irradiations for tumour cooling. Tumour size was measured every two days using a vernier calliper for 10 days after the first PDT treatment. The tumours were measured for the maximum width (*X*) and length (*Y*) and the tumour volumes (*V*) were calculated using the formula: *V*=(*X*^2^*Y*)/2. Changes in tumour volume as a function of time were determined for each mouse by normalizing the tumour volume at day *X* to the respective tumour volume at day 0 after treatment. Mice were randomly selected in each group, and the tumours were photographed at day 0, 5 and 10. At 10 days after treatment, the mice in each group were killed, and the tumours were removed, photographed and weighed.

### Statistical analysis

Statistical analysis was performed by two-sided Student's *t*-test for two groups, and one-way analysis of variance for multiple groups. A value of *P*<0.05 was considered statistically significant.

## Additional information

**How to cite this article:** Cheng, Y. *et al*. Perfluorocarbon nanoparticles enhance reactive oxygen levels and tumour growth inhibition in photodynamic therapy. *Nat. Commun.* 6:8785 doi: 10.1038/ncomms9785 (2015).

## Supplementary Material

Supplementary Information Supplementary Figures 1-8, Supplementary Table 1 and Supplementary References

Supplementary Data 1 Raw data for Fig5c_Intratumoral injection

Supplementary Data 2 Raw data for Fig6c_Intravenous injection
