# Supplementary material for: Perfluorocarbon nanoparticles enhance reactive oxygen levels and tumour growth inhibition in photodynamic therapy
Source: Nat Commun. 2015 Nov 3;6:8785. doi: 10.1038/ncomms9785 (PMC4659941; doi:10.1038/ncomms9785)
Supplement: Supplementary Information — Supplementary Figures 1-8, Supplementary Table 1 and Supplementary References [file ncomms9785-s1.pdf]

SUPPLEMENTARY INFORMATION

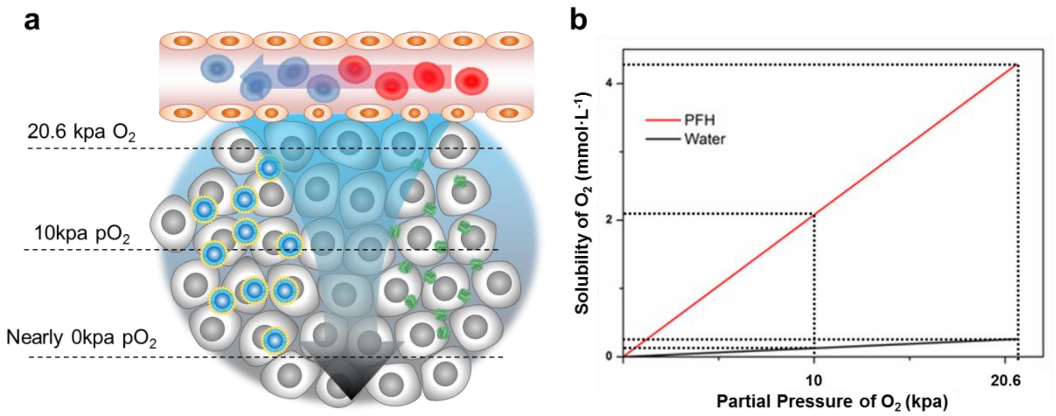

Supplementary Figure 1 Schematic of potential Oxy-PDT mechanisms *in vivo*. (**a**) Distribution of PDT agents in tumor microenvironment by EPR-effect, and the pO_2 gradient from well-oxygenated blood to hypoxic tumor center. (**b**) Relationship between oxygen solubility and pO_2 in PFH[1] and water. The dissolved oxygen in Oxy-PDT agents is always higher than that in tumor matrix at the same pO_2, due to the higher oxygen solubility of PFH.

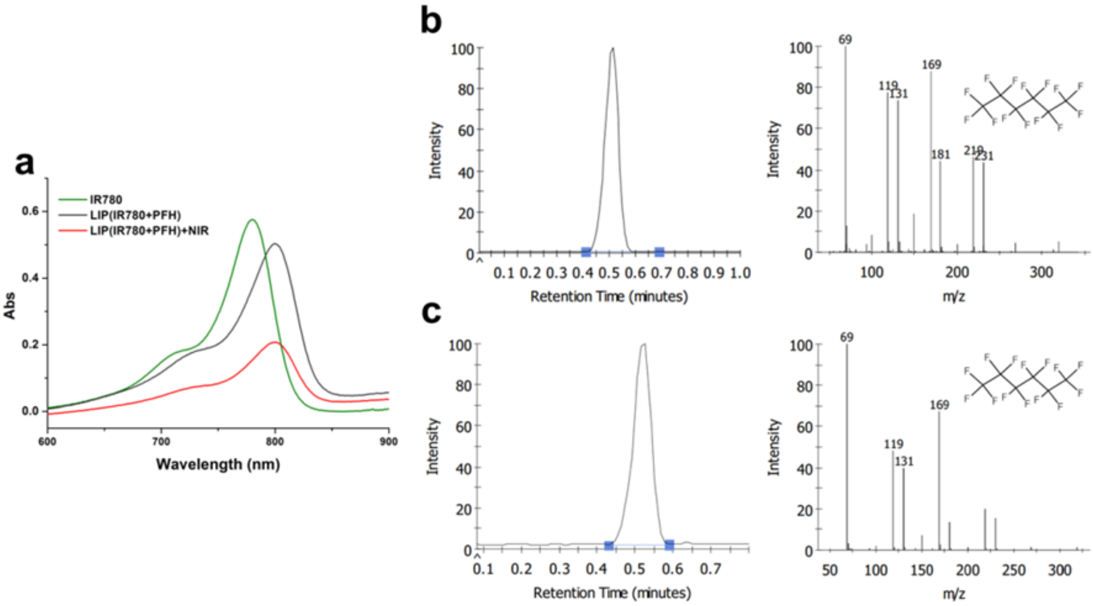

Supplementary Figure 2 （**a**）UV-vis spectra of IR780, LIP(IR780+PFH) and LIP(IR780+PFH) after laser irradiation (2 W per cm^2) at 808-nm for 20 s. （b）（c）GC-MS of PFH and LIP(IR780+PFH)

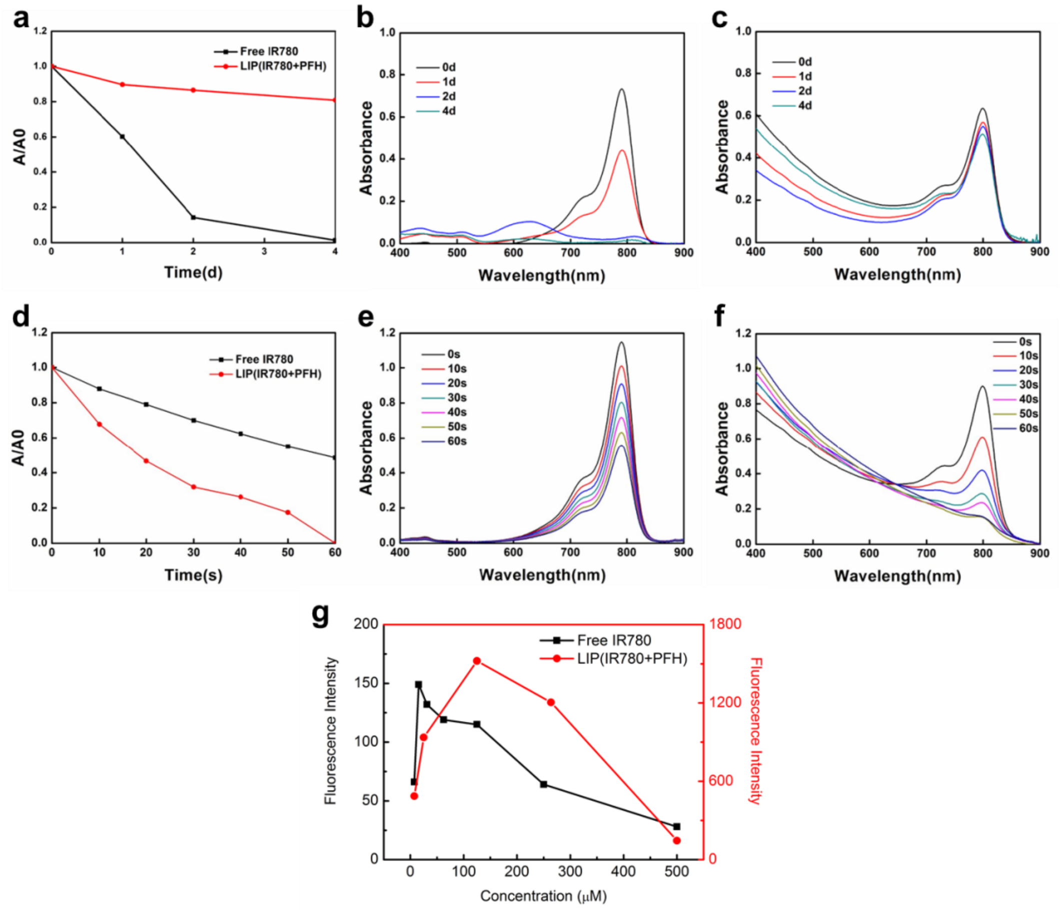

Supplementary Figure 3 (**a**) Normalized absorption of free IR780 and LIP(IR780+PFH) stored in dark. (**b**) UV-vis absorption spectra of **(b)** free IR780 and **(c)** LIP(IR780+PFH) stored in dark. (**d**) Normalized absorption of free IR780 and LIP(IR780+PFH) after laser irradiation (808nm 2W per cm^2). UV-vis absorption spectra of (**e**) free IR780 and (f) LIP(IR780+PFH) after laser irradiation (808nm 2W per cm^2). (**g**) Relationship between $^1O_2$ generation and concentration of free IR780 or LIP(IR780+PFH). The amount of generated $^1O_2$ was determined by the accumulated fluorescence intensity of oxidized SOSG (an indicator of $^1O_2$).

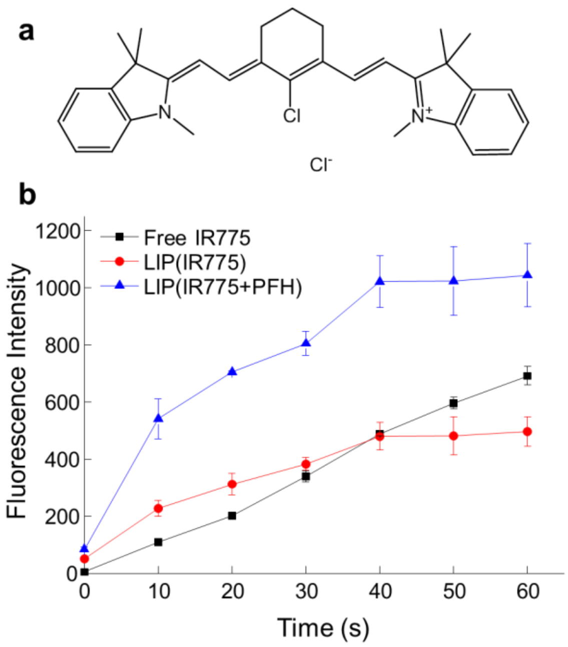

Supplementary Figure 4 Enhancement of $^1O_2$ generation of IR775 by Oxy-PDT. (**a**) Structure of IR775. (**b**) Comparison of $^1O_2$ generation between LIP(IR775+PFH) (16.7 µg per ml IR775, 30 v/v% PFH), and LIP(IR775) (16.7 µg per ml IR775), as determined by the accumulated fluorescence intensity of oxidized SOSG (an indicator of $^1O_2$). Fluorescence intensity (excited at 504 nm and measured at 525 nm) was plotted as a function of exposure time to a NIR laser (808-nm, 2 W per cm^2). Values are means ± s.d. (*n*=3).

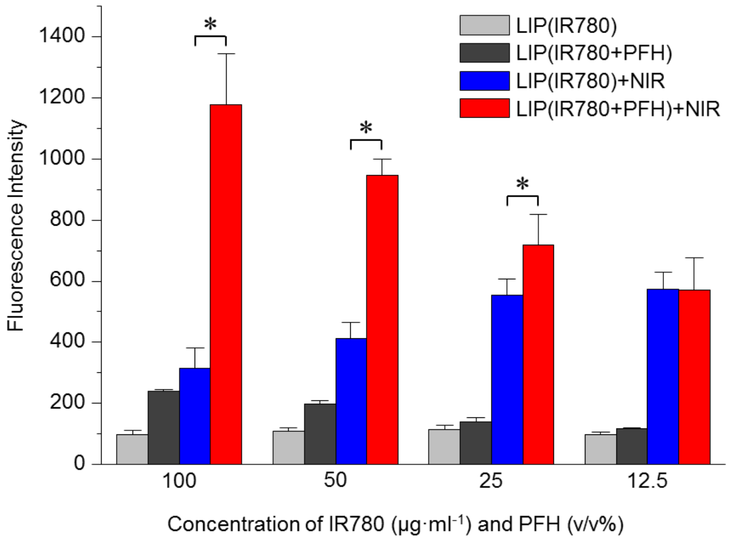

Supplementary Figure 5 $^1O_2$ production in concentrated solution. Fluorescence intensity of oxidized SOSG (an indicator of $^1O_2$, excited at 504 nm and measured at 525 nm) is plotted as a function of concentration. Values are means ± s.d. (n=3). Irradiation was given by 808-nm laser (2 W cm^{-2}) for 20 s. *P<0.05, one-way ANOVA.

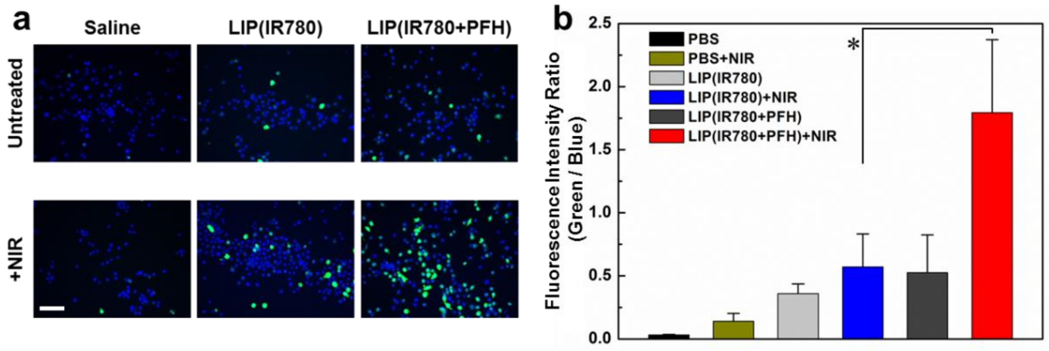

Supplementary Figure 6 Enhanced $^1O_2$ generation by Oxy-PDT on CT26 colon carcinoma cells. (**a**) ROS generated in cells treated with different agents and/or exposed to 808-nm laser (2 W per cm^2 for 20 s). Confocal images showed green fluorescence of carboxy-H_2DCFDA indicating positive staining for ROS; the positions of the cells are indicated by blue fluorescence indicative of nuclear counterstaining with Hoechst 33342 (scale bars, 50 μm). (**b**) Statistical analysis of average ROS amount per cell. The fluorescence intensity ratio was calculated by green fluorescence from carboxy-H_2DCFDA divided by blue fluorescence form Hoechst 33342 (n=3, *p < 0.05, v.s. LIP(IR780)+NIR, two-sided Student's t test).

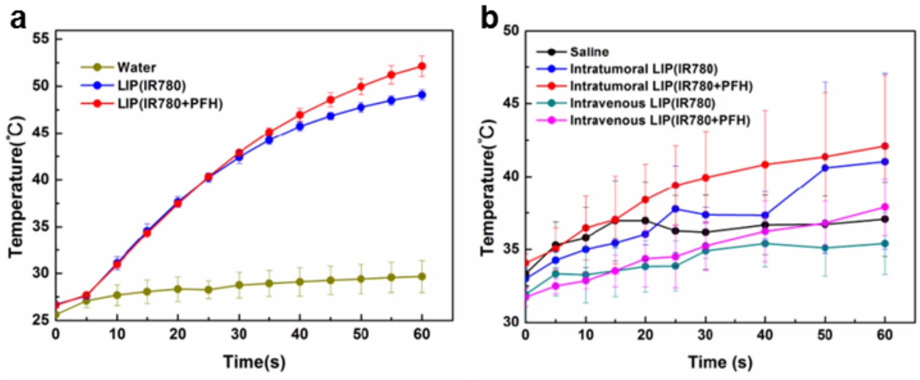

Supplementary Figure 7 Photothermal behaviors during Oxy-PDT. (**a**) *In vitro* heating up curves at a maximal concentration (16.7 μg per ml IR780, 50 v/v‰ PFH, 120 μl) higher than those used in all cellular experiments, measured by resistance thermometer. Values are means ± s.d. (n=3). (**b**) Heating up curves of tumors on mice treated with intratumoral administration (156 μg per ml IR780, 30 v/v% PFH, 50 μl) and intravenous administration (60 μg per ml IR780, 10 v/v% PFH, 200 μl) measured by infrared thermometer. Values are means ± s.d. (n=3).

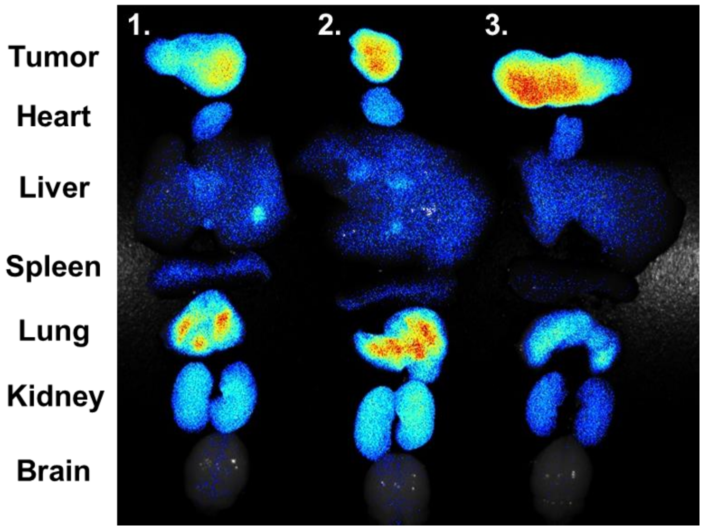

Supplementary Figure 8 Accumulation of LIP(IR780+PFH) in tumor. Biodistribution of IR780 in LIP(IR780+PFH) (0.2 ml, 60 μg per ml IR780, 10 v/v% PFH) at 24h measured by NIR imaging (*n*=3).

Supplementary Table 1 Conditional singlet oxygen quantum yield*

Photosensitizers	Φ_{so}
Ce6	0.65[a]
IR780	0.127
LIP(IR780)	0.078
LIP(IR780+PFH)	0.648

* These data were measured conditionally and involved approximations, do not use as the standard.

[a]: Reference data[2].

Supplementary References

1. Dias, A.M.A., Freire, M., Coutinho, J.A.P. & Marrucho, I.M. Solubility of oxygen in liquid perfluorocarbons. *Fluid Phase Equilibr* **222**, 325-330 (2004).
2. Ho, C.J., *et al.* Multifunctional photosensitizer-based contrast agents for photoacoustic imaging. *Scientific reports* **4**, 5342 (2014).
